# Hematopoietic stem cell transplant does not prevent neurological deterioration in infants with Farber disease: Case report and literature review

**DOI:** 10.1002/jmd2.12008

**Published:** 2019-03-14

**Authors:** Catherine Goudie, Abdulfatah M. Alayoubi, Pauline Tibout, Michel Duval, Bruno Maranda, David Mitchell, John J. Mitchell

**Affiliations:** ^1^ Division of Hematology‐Oncology, Department of Pediatrics McGill University Health Center Montreal Quebec Canada; ^2^ Division of Medical Genetics, Department of Human Genetics McGill University Montreal Quebec Canada; ^3^ Department of Biochemistry and Molecular Medicine, College of Medicine, Taibah University Madinah Saudi Arabia; ^4^ Department of Pediatrics CHU de Québec—Université Laval Quebec Québec Canada; ^5^ Division of Hematology‐Oncology, Department of Pediatrics CHU Sainte‐Justine, University of Montreal Montreal Quebec Canada; ^6^ Division of Genetics, Department of Pediatrics Université de Sherbrooke Sherbrooke Quebec Canada; ^7^ Department of Pediatrics McGill University Montreal Quebec Canada

**Keywords:** acid ceramidase, ASAH1, Farber disease, hematopoietic stem cell transplant, lysosomal storage disorder, macrophage

## Abstract

Farber disease (FD) is an inherited autosomal recessive disorder of lipid metabolism. The hallmark of the disease is systemic accumulation of ceramide due to lysosomal acid ceramidase deficiency. The involvement of the central nervous system is critical in this disorder leading to rapid deterioration and death within a few years after birth. Efforts to treat patients by hematopoietic stem cell transplant (HSCT) have resulted in favorable results in the absence of neurological manifestations. We report the outcomes of HSCT in two patients with FD who received early HSCT and had neurological deterioration posttransplant. We also present a new understanding of the limitations of HSCT in FD management based on our observations of the clinical course of the two patients after therapy.

## 1 INTRODUCTION

Farber disease (FD) (Online Mendelian Inheritance in Man #228000) is a rare but devastating lysosomal storage disorder (LSD) characterized by deficient acid ceramidase (ACDase) activity.^1^ Patients carrying two mutant alleles classically present with a triad of symptoms shortly after birth: deforming arthritis, subcutaneous nodules over the joints and pressure areas, and hoarseness of the voice. Involvement of the visceral organs—including the liver—has also been described.^2^ When the central nervous system (CNS) is involved, patients may have significant motor and cognitive impairments leading to rapid deterioration and death.^3^ Patients with an attenuated form of FD have peripheral manifestations and longer life spans in the absence of CNS involvement.^4^ As of yet, no genotype‐phenotype correlations have been established.^5^

The available treatment for FD is directed toward the management of the arthritis and associated pain through the use of antiinflammatory agents. Hematopoietic stem cell transplant (HSCT) has been attempted in some patients with variable results.^6^, ^7^, ^8^, ^13^ Patients with neurological involvement continued to deteriorate after HSCT while peripheral manifestations resolved.^9^, ^10^ The correction of CNS manifestations by HSCT has been reported in other neuropathic LSDs, with better treatment outcomes in some, but not all, LSDs if this procedure was performed prior to the development of neurological symptoms.^11^

We report two cases of children with FD who underwent HSCT before 1 year of age and who had neurological deterioration posttransplant. We believe this report is of significant importance to confirm that early HSCT during infancy is ineffective in preventing the CNS damage associated with FD. We also provide a new hypothesis that explains the limitations of HSCT in the management of FD.

## 2 CASE REPORT

### 2.1 Patient #1

A 3‐month‐old girl came to medical attention because of apparent pain, irritability, and difficulty with mobilization. Swelling and erythema over her interphalangeal joints were noted and the patient was referred to a rheumatologist who confirmed polyarthritis affecting her hands and wrists. The patient was also found to have a hoarse voice. Further investigations revealed signs of right vocal cord paresis and gastroesophageal reflux. She was the second child to a nonconsanguineous healthy caucasian couple, born at term with normal birthweight and height.

At the age of 4 months, erythematous nodules progressed over multiple areas of her body. The diagnosis of FD was suspected and she was found to carry a homozygous mutation (c.458A > G [p.Tyr153Cys]) in the *ASAH1* gene.

Initial neurological assessment revealed signs of mild delay in her gross and fine motor skills presumed to be secondary to arthritis. To further assess her neurological status, she underwent electroencephalography, which was unremarkable, and electromyography (EMG), which showed no evidence of peripheral neuropathy or myopathy. A decision was made for the child to undergo HSCT at the age of 8 months. Magnetic resonance imaging (MRI) done prior to transplant was unremarkable. Her initial ophthalmologic examination 4 months prior to transplant was normal. One cherry‐red spot was identified on reassessment a few days prior to transplant.

She received a myeloablative conditioning regimen with targeted IV busulfan, fludarabine, and alemtuzumab, followed by a matched (6/6) unrelated cord blood transplant at the age of 9 months. Cyclosporin and mycophenolate mofetil (MMF) were used as graft vs host disease (GvHD) prophylaxis. The patient had no significant transplant‐related complications and had full recovery of all three cell lines. No signs of acute or chronic GvHD were reported. Chimerism at day +475 was 100% donor cells.

**Figure 1:**
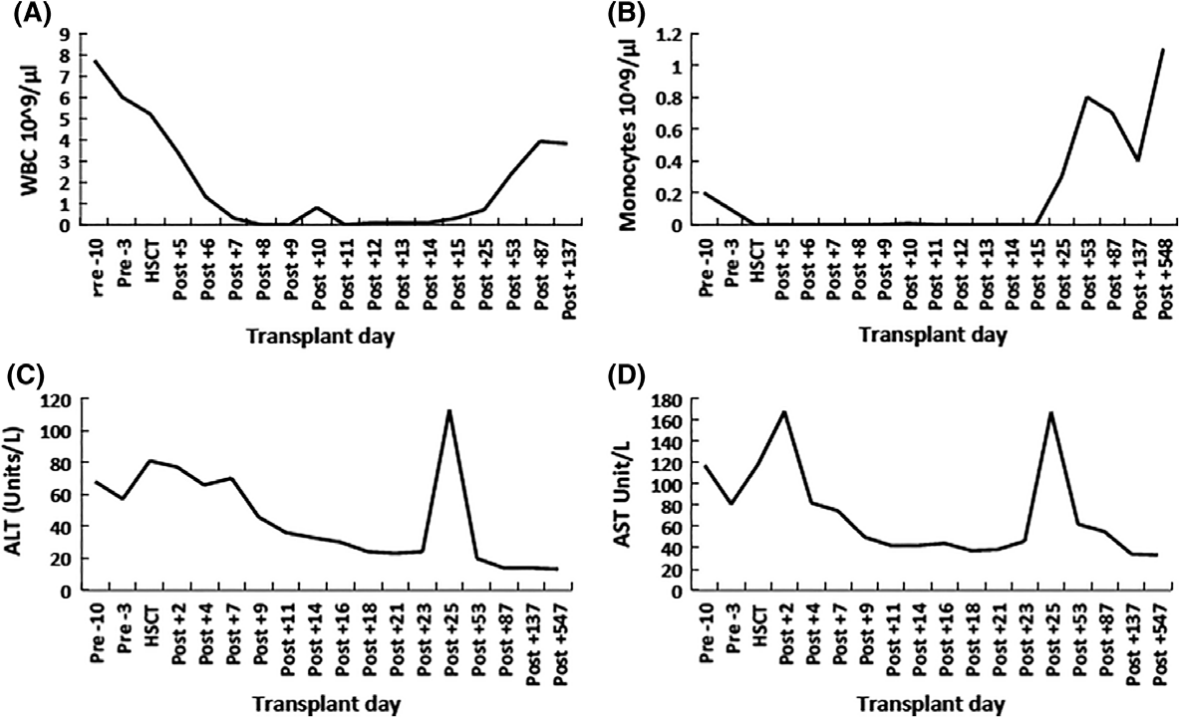
Correction of liver enzymes corresponds to the drop of peripheral leukocytes levels during conditioning and post‐HSCT. A, Peripheral blood levels of WBC and monocytes pre‐ and post‐HSCT. B, Plasma levels of ALT and AST pre‐ and post‐HSCT. ALT, alanine aminotransferase; AST, aspartate aminotransferase; HSCT, hematopoietic stem cell transplant; WBC, white blood cells

Of note, her alanine aminotransferase (ALT) and aspartate aminotransferase (AST) were elevated preceding the transplant (Figure 1). On day +7, ALT and AST started to decrease toward normal levels, with the exception of an unexplained spike on day +25. Subsequent values remained within normal limits (Figure 1).

In the first 12 months following HSCT, resolution of the skin nodules and significant improvement of her joint mobility were observed. Her arthralgia and arthritis resolved and pain and antiinflammatory medications were discontinued. Her gross and fine motor skills improved rapidly although she remained developmentally delayed for her age. Ophthalmological evaluation 6 months post‐HSCT identified the same cherry‐red spot with no change compared with previous examination.

At the age of 1‐year‐old (3 months post‐HSCT), a computed tomography scan demonstrated corticosubcortical bilateral frontotemporal volume loss, hypodensity of the brain parenchyma, and mild ventriculomegaly. She began to show signs of hypertonicity and irritability 12 months after HSCT with progressive loss of certain previously acquired skills. The following months were highlighted by the deterioration of her neurological status, increased autonomic dysfunction with severe constipation, and swallowing and feeding difficulties. The patient had multiple respiratory infections for which she required hospitalizations and admissions to the intensive care unit. She died of respiratory failure at the age of 37 months, 28 months after transplant.

### 2.2 Patient #2

A term male infant of nonconsanguineous healthy Caucasian parents was noted to have a hoarse voice since birth and painful occipital nodules appearing at 10 days of age. By 4 months of age, he developed multiple progressive painful nodules over his fingers, wrists, ankles, sternum, and back and lumbar areas. The clinical diagnosis of FD was made based on the classical clinical manifestations. Genetic workup identified a homozygous genotype for the same mutation in *ASAH1* (c.458A > G [p.Tyr153Cys]).

Initial neurological assessment at the age of 5 months identified motor developmental delay with axial hypotonia. MRI showed a prominent ventricular system, bilateral prominent arachnoid sulci, and normal parenchyma at this age. Demyelinating‐type conduction abnormalities involving motor and sensory nerves were noted on EMG at the age of 7 months. Reassessment of his neurological status at the age of 6 months revealed signs of upper motor neuron dysfunction including brisk tendon reflexes, clonus, and spasticity. Retinal cherry‐red spots were reported on ophthalmological assessment.

At 7 months of age, thickened vocal cords, edema, and severe erythema of the arytenoids were reported on laryngoscopy. He had poor weight gain and microcytic anemia. The patient had normal kidneys and no hepatosplenomegaly.

The child underwent a matched (6/6) unrelated cord blood transplant at the age of 9 months following myeloablative conditioning with targeted IV busulfan, cyclophosphamide, and antilymphocyte globulin. A rapid resolution of the painful nodules was observed following the initiation of the conditioning chemotherapy. The patient experienced major improvements of joint stiffness and better mobility. The child did not develop any significant transplant‐related complications and had full recovery of all three cell lines. Cyclosporin and MMF were used as GvHD prophylaxis. No signs of acute GvHD were reported. Chimerism studies showed 100% donor cells at 4 years posttransplant.

At 9 months posttransplant, a brain MRI showed stable findings compared with the pretransplant study. At 15 months posttransplant, signs of neurological deterioration became visible with loss of previously acquired motor milestones. A repeat MRI revealed progression in the dilatation of the ventricular system with cerebral parenchymal atrophy. The spine MRI was unremarkable. A video fluoroscopy swallowing study confirmed aspiration with an absent cough reflex. His overall clinical status continued to deteriorate with frequent hospitalizations for respiratory complications. The child died at 8 years old of respiratory insufficiency secondary to aspiration pneumonia.

## 3 DISCUSSION

Previous reports on HSCT for patients with FD showed variable outcomes (Table 1). Favorable results were observed post‐HSCT in patients without neurological manifestations^6^, ^8^, ^12^, ^14^ while progression of the disease continued to occur if the CNS was involved pre‐HSCT^9^, ^10^ or after transplant.^7^, ^13^

**Table 1:** Outcomes of hematopoietic stem cell transplant in Farber disease. Previous reports describing variable outcomes in patients with Farber disease treated with HSCT

Patient's age at HSCT	CNS involvement	Manifestations prior to HSCT	Clinical course post‐HSCT	Survival	References
18 months	+ (pre‐HSCT)	Neurological and peripheral manifestations	Progressive neurological deterioration	DOD 16 months post‐HSCT	9
9.5 months	+ (pre‐HSCT)	Neurological and peripheral manifestations	Regression of peripheral symptoms; deterioration of neurological status	DOD 28 months post‐HSCT	10
3 years and 11 months	−	Triad^a^ of symptoms, no neurological deficits	Resolution of arthritis and nodules	Alive at report, time not defined	8, 12
3 years and 10 months	−	Triad^a^ of symptoms, no neurological deficits	Resolution of arthritis and nodules	Alive at report, time not defined	8, 12
2 years	−	Triad^a^ of symptoms, no neurological deficits	Regression of arthritis and resolution of nodules	Alive at report, time not defined	12
21 years	−	Triad^a^ of symptoms, no neurological deficits	Improvement in joint mobility and resolution of nodules	Alive at report, time not defined	12
18 months	+ (post‐HSCT)	Triad^a^ of symptoms, failure to thrive, no apparent neurological deficits	Regression of arthritis and nodules, resolution of hoarseness. Neurological deterioration visible 12 months post‐HSCT	Alive 22 months post‐HSCT	7, 13
9 years	−	Arthritis, nodules, and bone destruction of the odontoid process with spinal cord compression	Resolution of nodules, improvement of joint mobility, marked improvement of bone structure of the odontoid process without further spinal compression	Alive 5 years post‐HSCT	14

Abbreviations: CNS, central nervous system; DOD, died of disease; FD, Farber Disease; HSCT, hematopoietic stem cell transplant.

a Triad of symptoms: hoarse voice, subcutaneous nodules, and arthritis.

We present in this report the outcome of HSCT in two unrelated 9‐month‐old patients of French Canadian descent with FD who had the same novel homozygous mutation in *ASAH1*, indicating the possibility of a founder effect. Patient 1 had very subtle clinical signs of CNS involvement prior to transplant, with evidence of a cherry‐red spot, while patient 2 manifested neurological dysfunction (including central and peripheral nervous system involvement) with abnormal brain MRI. Both the patients were transplanted under 1 year of age and maintained 100% chimerism of donor cells in the peripheral blood for extended periods. After the initiation of the conditioning for HSCT, subcutaneous nodules rapidly resolved and major improvements in joint pain and stiffness were observed resulting in a significant improvement in quality of life for the patients and their families.

Despite the minimal apparent CNS involvement prior to transplant and the high donor chimerism posttransplant, patient 1 developed CNS abnormalities evident on brain imaging and later manifested clear neurological deficits. Patient 2 continued to show progression of his neurological impairments with no signs of recovery after HSCT. Both children manifested swallowing difficulties with aspiration. This finding could be attributed to CNS impairment, decreased gastrointestinal motility, or peripheral nerve damage, which has been reported in FD. Both patients ultimately died of respiratory failure likely secondary to repeated aspiration. It is possible that their life span may have been prolonged. It was shown in the monocyte chemoattractant protein 1 (MCP‐1)‐deficient Farber mouse model that the reduction in the peripheral tissue damage resulted in an increase in the life span despite unchanged CNS defects.^15^

Although the general assumption that the correction of the disease phenotype by HSCT is attributed to the donor leukocytes,^5^ we have observed that the initial amelioration of disease (non‐CNS) manifestations may be secondary to the elimination of the host ACDase‐deficient white blood cells (WBC) rather than the delivery of leukocytes with normal enzyme activity. It was suggested that overexpression of ACDase in the donor bone marrow is needed to achieve metabolic cooperativity.^16^ Transduced fibroblasts overexpressing ACDase from FD patients secrete a large excess of ACDase, while secretion from normal fibroblasts is minimal.^17^

The clinical assessments of patient 2 documented the resolution of subcutaneous nodules after initiating the myeloablative conditioning prior to delivering the donor hematopoietic stem cells. In addition, liver enzyme levels (AST and ALT) from patient 1 corrected when the peripheral WBC counts reached their nadir post‐HSCT. These findings make it reasonable to suggest that ACDase‐deficient leukocytes (likely macrophages) are responsible for the tissue damage seen in FD. By eliminating abnormal ACDase‐deficient macrophages, physically, through the conditioning therapy or functionally by providing these cells with normal ACDase activity through gene or enzyme replacement therapies,^18^, ^27^ the tissues can be protected. It seems important to note the distinction between bone‐marrow‐derived monocytes and tissue‐resident macrophages in the pathophysiology of FD. MCP1‐mediated tissue infiltration by monocytes was suggested as an underlying mechanism to explain the tissue damage seen in FD.^18^ Although MCP1 deletion resulted in a partial reduction of infiltrations and delayed progression of the disease in the peripheral tissues, the damage continued to occur in these organs and the CNS defects were unchanged.^15^ Also of interest, the organs that largely showed infiltrations in the Farber mouse model are the sites of the traditional reticuloendoethelial system, which is comprised of tissue macrophages.^18^ These observations along with the finding that myeloablation does not prevent CNS deterioration indicate that the major damage seen in FD does not result from circulating monocytes. Resident macrophages could be playing a major role in FD pathogenesis independent of bone‐marrow‐derived monocytes.

The question remains as to why HSCT is effective in protecting the peripheral organs in FD but not the CNS. Accumulating data has shown that a population of tissue‐resident macrophages is maintained independently of circulating monocytes.^19^ After exposure to genotoxic injury followed by HSCT, the majority of host tissue macrophages are derived from donor bone‐marrow hematopoietic precursors.^19^ This competitive replacement ensures effective elimination of host ACDase‐deficient tissue macrophages, thereby sustaining the protection of organs against the damage imposed by host cells. In the CNS, lipid‐laden macrophages were observed in areas that were largely damaged including spinal cord tracts and CNS white matter.^18^, ^20^ The replacement of tissue‐resident macrophages in the CNS by donor monocytes is incomplete^21^ and predominantly limited to perivascular and leptomeningeal sites^22^ and may take years.^23^ Although HSCT during the newborn period was found to enhance donor‐cell brain engraftment in some LSD animal models, the number of brain‐engrafted cells remained very low.^24^ Depletion of host microglia is critical for the reconstitution of brain macrophages by donor cells.^25^ The inefficient replacement of host tissue macrophages in the CNS enables these cells to regenerate post‐HSCT, as they are capable of self‐renewal.^26^ The persistence of ACDase‐deficient microglia, rather than the absence of normal macrophages, may explain the progression of neurological deficits in patients with FD despite HSCT.

Our data corroborate the previous findings that HSCT is ineffective in preventing disease progression in patients with FD who have CNS involvement. We further showed that HSCT during infancy was ineffective in protecting against the CNS damage in the patient who had minimal clinically objectified neurological abnormalities prior to transplant. We also present a new insight on the role of the host macrophages in the outcome of HSCT.

## AUTHOR CONTRIBUTIONS

Catherine Goudie and Abdulfatah M. Alayoubi were involved in all aspects of the manuscript and case report. Pauline Tibout collaborated in the collection of medical data regarding the case reports. Bruno Maranda, David Mitchell, and Michel Duval reviewed the manuscript and provided insight and expertise for this case report. John J. Mitchell supervised the study, reviewed, and edited the manuscript, and provided directions throughout the work on this report.

## CONFLICT OF INTEREST

There are no conflicts of interest to declare.

## ETHICS APPROVAL

REB approval was obtained at the Montreal Children's Hospital and at CHU de Québec‐Université Laval to access the medical charts.

## PATIENT CONSENT STATEMENT

Both patients' parents have consented to the revision of the medical charts and presentation of this manuscript.
